# Impact of Salts Mixtures on the Physicochemical and Sensory Characteristics of Spanish-Style Manzanilla Green Table Olives during Packaging

**DOI:** 10.3390/foods12193561

**Published:** 2023-09-25

**Authors:** Antonio López-López, José María Moreno-Baquero, Antonio Garrido-Fernández

**Affiliations:** Instituto de la Grasa (IG), CSIC, Campus Universitario Pablo de Olavide, Edificio 46, Ctra. Utrera km 1, 41013 Sevilla, Spain; jose.moreno.baquero@gmail.com (J.M.M.-B.); garfer@cica.es (A.G.-F.)

**Keywords:** table olive packaging, sodium substitution, calcium, potassium, magnesium, physicochemical characteristics

## Abstract

Using response surface methodology (RSM), this study investigates the effect of NaCl substitution (50%) with KCl, CaCl_2_, and MgCl_2_ in the packaging brines (controlled variables) on the characteristics (responses) of plain green Spanish-style Manzanilla olives, maintaining the salt-mixture level of 5%. The RSM showed that the increment of CaCl_2_ caused a linear significant (*p*-value ≤ 0.05) decrease in pH and a linear increase in *firmness* (instrumental), *hardness* (panel scores), and *crunchiness*. The models for *bitterness* and *fibrousness* also included quadratic (CaCl_2_·MgCl_2_) and cubic (the three salt) interactions, which led to areas of minimum and maximum scores around the central points of the CaCl_2_-MgCl_2_ and KCl-MgCl_2_ axes, respectively. In contrast, the increase in the KCl level linearly decreased *bitterness* scores. Optimisation resulted in a relatively low desirability (0.57) and the selection of a combination that may necessitate further refinement, such as increasing KCl or reducing CaCl_2_ levels, especially for markets sensitive to *bitterness*. Interestingly, the *overall score* and *buying predisposition* positively correlate with *salty*, *smell*, *acid*, and *appearance* and negatively with *bitterness*. Furthermore, PLS-R analysis found that the pivotal attributes influencing *overall appreciation* were *smell* and *crunchiness* while *buying predisposition* was promoted by *crunchiness*. Conversely, *bitterness* had a detrimental impact on these appreciations. Cluster analysis grouped the experimental runs into four categories, with sensory profiles predominantly diverging in *bitterness*, *salty*, and kinesthetic characteristics. Ultimately, this study elucidates four distinct sensory profiles that consumers experience.

## 1. Introduction

Table olive production has progressively increased during the past decades, expanding beyond the typical initial use in the Mediterranean countries such as Greece, Syria, Algeria, Italy and Spain. This expansion has extended to countries worldwide, including the USA, Argentina, Perú, and Australia) [1]. This growth has been fueled by the introduction of larger fermentation/storage containers (16 tonnes) and the mechanisation of conditioning operations like pitting, stuffing, or slicing. Additionally, table olives, as a component of the Mediterranean diet, are gaining acceptance in other non-producing countries such as Canada, Brazil or Russia. Global production and consumption currently reach about 3 × 10^6^ tonnes [1].

Traditionally, sodium chloride (salt) has been the main component in brines used for fermentation/storage and packaging [2]. Its presence in the current state-of-the-art processing technology is essential for preventing sanitary risks, characteristic taste, and appropriate pH levels. A mineral content survey of Spanish cultivars [3] revealed that the sodium content in the most popular olive presentations falls within the following ranges, expressed in g/100 g olive pulp: for green Spanish-style, 1.44 (Hojiblanca)–1.72 (Gordal); for ripe olives, 0.58 (Gordal)–0.94 (Manzanilla); and for directly brined olives, 1.5 (Manzanilla)–1.67 (*Aloreña de Málaga*). Consequently, considering that the green Spanish-style olives constitute around 50% of production, they contribute the most to sodium intake for table olive consumers. In contrast, ripe olives (accounting for 45% of consumption) contribute the least.

The link between sodium intake and cardiovascular issues is well-established. Recent reviews have delved into the relationship between sodium intake, the risk of cardiovascular diseases and their dose-response correlation [4]. The meta-analysis encompassing 36 reports and 616,905 participants concluded that individuals with high sodium intake faced a 1.19-fold higher adjusted risk of cardiovascular disease than those with low sodium intake. Analysing 20 of these reports for a dose-response investigation, a significant linear connection between dietary sodium intake and cardiovascular risk emerged, showing a 6% increase in risk for every 1 g of dietary sodium increment. This mounting concern surrounding sodium (or salt) ingestion is reflected in recommended intakes. The EU sets the reference intake at 2.4 g Na/day [5], and the Dietary Guidelines for Americans limit it to 2.3 g Na/day [6]. Comparable recommendations are seen in other countries. In the EU, strategies to reduce salt levels in the diet have been outlined [7], primarily targeting frequently consumed salt-rich foods (like bread or meat products), although not table olives due to their minor contribution to the EU diet.

In the context of table olives, the interest in reducing salt levels in table olives has parallel consumer concern. However, up to this point, most efforts have primarily focused on the fermentation/storage phases of the most relevant cultivars. Özay and Borcakli [8] undertook research to modify the traditional fermentation process for naturally black olives. They tested brine concentrations of 14 g NaCl/100 mL (as usual) and 6 g NaCl/100 mL (reduced level) but found no sensory differences between the two conditions. However, this reduction in salt content led to a decrease in ash content from 4.7 g/100 g olive pulp to 2.41–2.81 g/100 g olive pulp. Tassou et al. [9] also employed various brine NaCl levels to process *Conservolea* naturally black olives. Brine levels of 6% and 4% in brines favoured the growth of lactic acid bacteria and prevented off-odour development.

Kanavouras et al. [10] conducted tests involving the total or partial substitution of NaCl (16%, *w*/*w*) with a buffer (CH_3_COOH, 0.05 M + Ca(OH)_2_, 0.025 M). They found that partial substitution resulted in a product with significantly better texture, colour and high acceptability. Tassou et al. [11] also investigated the effect of CaCl_2_ (5 g/L brine) on the mechanical properties and microbial characteristics of cv. *Conservolea* naturally black olives fermented at different sodium chloride concentrations (4, 6, and 8 g NaCl/100 mL). The pulp exhibited greater strength and stiffness when the calcium salt was added to the 4 g NaCl/100 mL brine. In *Aloreña de Málaga* cracked olives [12], fermentation using a mixture of Na, Ca, and K chloride salts, the behaviour of K was similar to Na, while the presence of Ca led to faster acidification, a lower pH and higher water activity. Panagou et al. [13] researched to examine the implications of NaCl reduction on the fermentation profile of *Conservolea* natural black olives, using five combinations of NaCl, KCl, and CaCl_2_. It was found that olives fermented with 4% NaCl and 4% KCl had good organoleptic attributes, while the addition of CaCl_2_ rendered the product bitter.

Concerning Spanish-style olives, Bautista Gallego et al. [14] employed various chloride salts, including sodium (in the range of 0–4%), potassium (0–4%) and calcium (0–6%) to ferment cv. *Gordal.* Adding CaCl_2_ reduced the pH, delayed sugar diffusion into the brine, and decreased yeast growth. In this fermentation process, the final product’s Na exhibited a linear relationship with the content in brine, while Ca and K contents followed quadratic models. Furthermore, most sensory attributes were linearly associated with the mineral contents in the brine, except for bitterness, which followed a quadratic model [15]. More recently, Dalloul and Erten [16] examined the physicochemical properties of cracked green cv. *Sari Ulak* olives fermented in brines where NaCl had been partially replaced with KCl, MgCl_2_, and CaCl_2_. The preferred olives were those prepared with NaCl, followed by NaCl + KCl and NaCl + MgCl_2_, whereas those containing CaCl_2_ were rejected because of their bitter taste.

Nevertheless, a more feasible avenue for salt reduction could lie in the packaging phase. This approach gains traction because the final products usually stabilise via pasteurisation, allowing for lower salt levels [17]. Furthermore, incorporating various chloride salts can enhance the daily intake of naturally occurring minerals like Ca, K, or Mg in fruits. This makes them more appealing from a health perspective and opens up the possibility of making health claims related to vitamin E or polyphenols [18].

However, such modifications might also have unintended repercussions on the sensory characteristics, making it crucial to thoroughly investigate the potential impacts on consumer-facing products.

This research investigated the impact of partial substitution (50%) of NaCl by KCl, CaCl_2_, and MgCl_2_ in the packaging brines of whole (plain) green Spanish-style Manzanilla table olives. The study focused towards examining the physiochemical and sensory properties of these olives. Throughout the experiment, the established salt level recommended by the Trade Standard Applying to Table Olives (5% NaCl in the packaging brine or juice after osmotic balance) was maintained [17] to preserve the traditional sensory characteristics of products. This approach aimed to simultaneously decrease sodium content and enhance potassium, calcium, and magnesium contents.

## 2. Materials and Methods

### 2.1. Olives and Experimental Design

The Manzanilla green olives, which have undergone processing following the traditional green Spanish-style during the 2018/19 season, were provided by JOLCA SA (Huevar, Sevilla, Spain). After fermentation, the olives were stored in a brine solution containing about 10% NaCl as they awaited packaging. For the packaging experiment, a batch of 20 kg of olives (size 240 fruits/kg) was transported to the facilities of the Instituto de la Grasa (IG), CSIC (Sevilla, Spain), where they were stored in a cold room until further use. The initial step involved a desalting process at 8 ± 1 °C, aimed at reducing the salt content to 2.5% (g/100 mL moisture of olive pulp), the targeted NaCl proportion for the final product. To this purpose, the olives (20 kg) were immersed in 32.75 L tap water for 72 h, a period considered enough to reach equilibrium based on previous desalting experiments. Before proceeding with packaging, samples of olives and brine in the same proportion as in the desalting containers were withdrawn to confirm the expected equilibrium. Other characteristics of the desalted olives included a pulp proportion of 84.58 g/100 g of olives and a moisture content of 76 g/100 g of olive pulp.

Subsequently, the low-sodium fruits were packaged in glass containers containing 170 g of olives and 130 mL of brine. As outlined in Table 1, various brine formulations were employed for this purpose. These brines consisted of a mixture of KCl, MgCl_2_, and CaCl_2_, ensuring their combined total sum was constrained to 25 g chloride salt mixtures/L brine (Table 1, expected levels in the customary units employed in table olive processing). The experimental design was generated using Design Expert 13.0 (Stat-Easy Inc. Minneapolis, MN, USA). However, due to the post-equilibrium of the concentration’s adjustments, the initial levels of nutrient salts in the cover brines were adjusted. The adaptation considered factors such as the hydration degree of the respective nutrient salts and the olive pulp/brine ratio in the containers. Regardless of the other salts, all the brines also included 2.5% NaCl to maintain equilibrium with the salt concentration in the pulp moisture of the desalted olives. Consequently, the total concentrations of the diverse salts in the brines remained consistently at 5 g/100 mL, aligning with the recommended level for lye-treated green table olives set by the International Olive Council’s Trade Standard for Table Olives [17]. Moreover, the equilibrium pH and lactic acid proportions were targeted to achieve 4.0 units and 5 g/L, respectively [17]. To assure product safety and quality, the glass containers were pasteurised at 85 °C for 8.5 min to reach a ${PU}_{62.4{}^{\circ}C}^{5.25}\geq$ 15, following prevailing practices within the table olive industries nowadays. Following pasteurisation, the containers were stored for two months at 20 ± 2 °C in the Instituto de la Grasa (IG), CSIC (Sevilla, Spain) pilot plant facilities. This period was designated to facilitate equilibrium attainment and simulation of the previous commercialisation of products in factory storage.

### 2.2. Physicochemical Analysis of Fruits

The brine pH, titratable acidity, and combined acidity were measured according to Garrido-Fernández et al. [2] using 25 mL of brine or pulp moisture. In short, the pH, titratable acidity and combined acidity analysis were made using Titroprocesor 670 (Methohn Instrumental, Inc., Herisau, Switzerland). The equipment read sequentially the pH, titratable acidity (by titration with 0.5 M NaOH solution to pH 8.0, and combined acidity by titration with a 2N HCl solution to pH 2.6. For the determination of lactic acid by HLPC, the method described by Sánchez et al. [19] was followed. Moisture was estimated by drying an aliquot of the olive pulp samples in still stainless plates till constant weight, using a Selecta electric oven at 106 °C (Dry-Big 2002970, Abrera, Barcelona, Spain).

Instrumental *firmness* was measured using a Kramer shear compression cell coupled to a universal testing machine (Instron, Canton, MA, USA). The cross-head speed was set to 200 mm/min. The *firmness* of the olives was the mean of 20 measurements, each of which was performed on one pitted fruit. Shear compression force was expressed as Newtons per gram (N/g olive pulp) [2].

The olive surface colour was determined using a Color-View spectrophotometer (model 9000, BYK-Gardner, Columbia, MD, USA) equipped with computer software to calculate the CIE coordinates: L (lightness), a* (negative values indicate green and positive values indicate red), and b* (negative values indicate blue and positive values indicate yellow). The apparatus was equipped with a C-type illuminant at 10°. Interference by stray light was minimised by covering samples with a box with a matt black interior. Each measurement recorded was the mean of 20 olives’ readings. Other colour measurements were the ratio –a*/b* (a kind of internal standardisation), habitually used to follow the green colour evolution in vegetables; the hue angle (*h*_ab_), the angular component of the polar representation (*h_ab_* = arctan b*/a*); and the chroma (*Ch*), the radial component [20,21]. Furthermore, the colour was also studied by the Colour Index (*Ic*) (*Ic* = (−2R_560_ + R_590_ + 4R_635_)/3, where the R values are reflectance at 560, 590, and 635 nm, respectively [22]. Sánchez, Rejano, and Montaño [22] summarised the relationship between *Ic* and the subjective colour appreciated by a panel in the scale shown in Table 2.

### 2.3. Sensory Analysis

The sensory analysis was conducted within individual booths under controlled light, temperature, and humidity conditions. The panel consisted of 100 experienced consumers from the Food Biotechnology Department staff. All of them were habitual consumers of green olives, familiar with table olive classification, had between 3 and 10 years of experience and had participated in other sensory studies of table olive presentations [23]. After a review of the literature and professional sources for descriptors [24], a modified version of the assessment sheet advocated by the Sensory Analysis for Table Olives [25] was embraced. The test consisted of two steps. In the first, the olives were checked for the absence of defects. Then, the olives having reached commercial classification were evaluated for *appearance*, *smell, acid, bitterness, salty, hardness, fibrousness, crunchiness, overall scoring, and buying predisposition*, previously used in other Descriptive Quantitative Analysis (DQA) of table olives [8]. Before each testing session, panellists received comprehensive information about the study’s objectives and the significance of the distinct descriptors. Such orientation sufficed for experienced subjects [24]. Samples were coded with a 3-digit random number and served in similar cups to those recommended in the Method for Sensory Analysis of Table Olives [26]. Only olives from four runs (including replicates) were concurrently presented to the panellists. This approach, implemented through a balanced, randomised order, aimed to eliminate potential presentation bias [27]. The panellists were asked to score the olives using a 10-cm unstructured scale. Anchor ratings were 0 (no perception) and 10 (extremely strong) for gustatory perceptions and low and high levels for kinaesthetic sensations [28]. *Overall scoring and buying predisposition* were evaluated on the same scale according to the global consumers’ appreciation. The responses from the questionnaire were quantified by measuring the distance (in 0.1 cm) from the left anchor. Subsequently, average scores for each descriptor and run were derived, processed, and employed as inputs for further analysis, including mixture design, clustering, and principal component analyses, following the framework described by Hibbert [29].

### 2.4. Effect of Mixture Composition on Physicochemical and Sensory Characteristics

The effect of KCl, MgCl_2_, and CaCl_2_ mixtures in the packing brines on the physicochemical and sensory attributes of the packaged table olives was systematically examined by applying response surface methodology (RSM). The experimental design was a simplex lattice cubic model, with the process for generating and interpreting these models detailed elsewhere [30]. This methodological approach involves several steps, guided by applying a Type II sequential model sum of squares. This process assesses the significance of the various terms within the response surface, culminating in the analysis of a possible special cubic model, mathematically defined by the following equation, expressed in the canonical (Sheffe’) form:

(1) $$R=\sum_{i=1}^{n}\beta_{i}x_{i}+\sum_{1\;\leq \;i<j}^{n}\beta_{ij}x_{i}x_{j}+\sum_{1\;\leq \;i<j<k}^{n}C_{ij}x_{i}x_{j}x_{k}+Є$$

where n represents the number of variables, namely x_1_, x_2_, and x_3,_ which correspond to the expected concentrations of KCl, MgCl_2_, and CaCl_2_ in the packaging brine after equilibrium, respectively; Є stands for error; R denotes the responses to be modelled (colour variables and sensory scores), and the *β* and *C* values are the coefficients to be estimated. The terms which, when added, significantly increased the proportion of variance were retained, and the end resulting model suggested. During the stepwise selection process, the criteria for entering and removing variables were set at *p* ≤ 0.05 and *p* ≤ 0.10, respectively. These different levels were motivated by the consideration that a term initially selected at *p* ≤ 0.05 could still enhance the model performance if retained at *p* ≤ 0.10. Subsequently, the model’s performance in terms of fit significance (*p* < 0.05) and non-significance (*p* > 0.05) of lack of fit was evaluated using ANOVA. Other parameters, such as adjusted R-square, precision, or standard errors of coefficients, were also scrutinised to assess the model quality. Finally, the equations, in terms of actual components (in g/L of the expected equilibrium packaging brine), were deduced and plotted to facilitate the interpretation of the models’ outcomes. The model design was selected and evaluated using Design-Expert v 13.0 (Stat-Easy Inc., Minneapolis, MN, USA).

### 2.5. Multivariate Analysis

Cluster analysis and PLS regression (PLS-R) were used to study the relationships between the diverse physicochemical parameters or treatments’ correlation. Correlation is a statistical measure that expresses the extent to which two variables are linearly related (e.g., they change together at a constant rate). It describes a simple relationship without making a statement about cause and effect. Hierarchical Cluster analysis (HCL) is a statistical technique that identifies groups of samples (or attributes) that behave similarly or show similar characteristics and thus quantify the structural elements of the samples or variables [31]. PLS-R is a method to relate two data matrices, X and Y, by a linear multivariate function, modelling the structures of X and Y. The usefulness of PLS-R derives from its ability to study data with many noisy (collinear) and even incomplete variables in both X and Y. This tool also improves the precision of the model parameters with the increasing number of relevant variables and observations. The approach is simple and constitutes a standard tool in chemistry and food technology [32,33]. The multivariate analysis used XLSTAT v 2017 (Stat-Soft, Paris, France).

## 3. Results

The fermented plain green olives used for the experiment had an average weight of 4.32 (standard error, 0.11) g. They were stored in a brine which, among other characteristics (Table 3), contained 93.7 g/L NaCl (salt).

### 3.1. Effect of the Desalting Operation and Partial Replacement of Salt on the Physicochemical Characteristics of the Product

The first target of the work was desalting the stored olives to reach 25 g NaCl/L in the pulp moisture. Beyond the NaCl leaching, a substantial proportion of titratable acidity, combined acidity, and estimated or determined by HPLC, lactic acid was also solubilised into the desalting solution (as outlined in Table 3). Interestingly, despite containing only half-combined acidity of the storage brine, the pH of the solution was slightly elevated, possibly because of its marked lower titratable acidity (Table 3). Regarding fruits, the desalinisation process reduced lactic acid presence to one-third of its initial level. Concurrently, moisture content increased by about 7%, although a portion of this increase was lost in most runs. Additionally, the *firmness* of the olives decreased during the process. However, this decrease was recovered during the subsequent packaging phase and even improved in some runs (as detailed in Table 3). The final *firmness* values were comparable to or even higher than those typically observed in commercial products of the same cultivar and style. Therefore, even at low temperatures (8 °C), the desalting process exhibited a degree of aggressiveness. Consequently, it should be performed carefully.

The concentrations of the salt mixtures modified these parameters across nearly all experimental runs (as depicted in Table 3). The initially targeted uniformity in titratable acidity, intended to be maintained throughout all treatments, exhibited a range from 1.5 to 3.30 g/L due to the varying presence of the added salts within each run. Following packaging, a drastic, consistently observed reduction in combined acidity was caused by diluting its residues into the new brine (Table 3). This decrease displayed notable variability, ranging from 14.2 to 24.9 mEq/L between different runs, each characterised by distinct salt mixtures (as detailed in Table 3). Still, the shift was favourable as diminished combined acidity corresponded with lower pH levels that are advantageous for preserving the product [2].

Furthermore, packaging also affected the olives’ instrumental *firmness*, which ranged from 15.39 to 22.72 N/g olive pulp. Notably, the maximum and minimum values were observed in runs containing the highest proportions of calcium (specifically runs 1 and 11) and in cases where calcium was absent (runs 3 and 8). Regarding moisture content, the packaging step reduced the increase in weight observed in desalting. The resultant moisture content ranged from 72.62 to 76.11 g/100 g olive pulp. Noticeably, at the point of packaging equilibrium, the sum of the titratable acidity and the lactate content from combined acidity (estimated as lactic) closely corresponded with the lactic acid concentration determined through HPLC analysis.

Nevertheless, the effect of the various packaging brines on the physicochemical characteristics (Table 3) can be studied in more detail by relating the concentrations in the salt mixtures (presented in Table 1) with the corresponding parameters (Table 3) through the utilisation of the RS technology. By treating each parameter as a response, the primary significant effect found was for pH. Additionally, although to a slightly lesser extent, a notable influence was also observed for instrumental *firmness*, which model exhibited proximity to significance and no significant lack of fit. Despite its limitations, the latter effect warrants consideration due to its impact on table olive quality. In addition to analysing individual models, giving the numerous variables under scrutiny, employing multivariate techniques becomes invaluable for exploring possible relationships between salt combinations and physicochemical characteristics or associating runs with similar features.

### 3.2. Effect of Partial Replacement of Salt on the Physicochemical Characteristics of the Products as Assessed by RSM

The model suggested relating the salt mixtures and pH was linear (*p*-value = 0.0384), significant (*p* = 0.0384) and had no lack of fit (*p* = 0.8655). The equation in actual explanatory variable units (g/L) was:

(2) $${pH}_{packaging}=+0.149\cdot \left[KCl\right]+0.113\cdot \left[{CaCl}_{2}\right]+0.133\cdot [{MgCl}_{2}]$$

**Figure 1:**
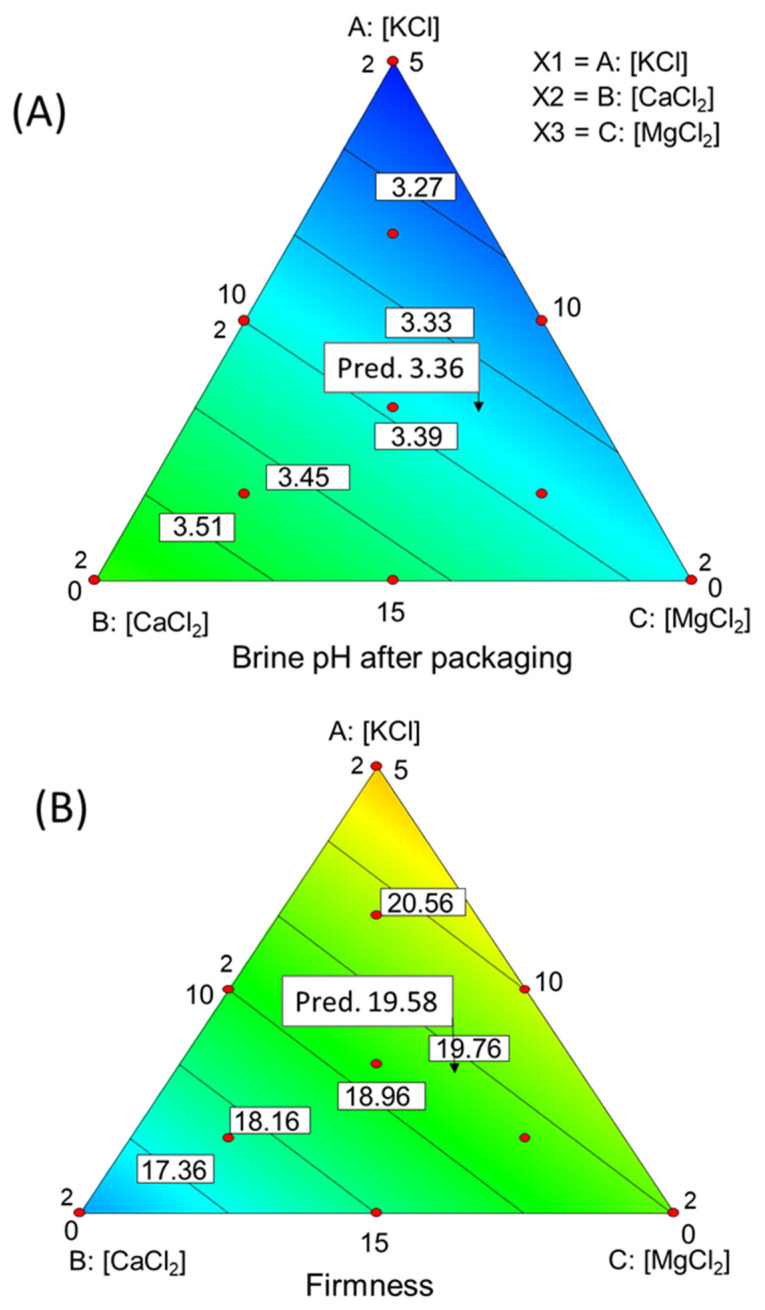
Impact of the CaCl_2_, KCl, and MgCl_2_ (g/L) mixtures on the (**A**) pH of the packaging brines and (**B**) instrumental *firmness* of olives. Pred., predictions at the optimisation point.

This equation could imply that KCl and MgCl_2_ are the salts contributing most significantly to pH elevation. However, it is important to note that interpreting the model, which may be linear, is not as straightforward within the simplex as in Euclidean space. Therefore, for a more practical understanding, it is advisable to visualise it (Figure 1A). It takes on a surface-like *appearance* when viewed in a 3D context, with contour lines offering a more intuitive comprehension. The contour lines exhibit a near-prependicular alignment with the CaCl_2_-KCl axis. This trend suggests that for each ratio (CaCl_2_/KCl) of these elements, there is a consistent pH level, regardless of the corresponding concentration of MgCl_2_.

Moreover, the pH decreases as the proportion of CaCl_2_ increases or, conversely, increases as the proportion of KCl rises. The diverse salt concentrations have a relatively narrow pH range of approximately 0.3 units. While seemingly modest, this range holds the potential for significant implications on the safety and stability of the product. This effect can be related to the affinity of Ca for the OH^-^ anions, leading to the subsequent release of H^+^ ions.

The model for instrumental *firmness* was significant at *p* ≤ 0.10 level (*p*-value = 0.0691), slightly higher than the usual threshold, and its lack of fit was not significant (*p*-value = 0.0753). The precision was 4.969 (values above 4 are considered appropriate). Although with convenient caution, it may be illustrative of the broad response of this parameter to the use of salt mixtures in table olives. Then, from the food technology viewpoint, the relationship is of great interest. The model was linear (*p*-value = 0.0691), and the equation in actual units was:

(3) $$Firmness=+0.599\cdot \left[KCl\right]+1.079\cdot \left[{CaCl}_{2}\right]+0.758\cdot \left[{MgCl}_{2}\right]$$

The function structure and the contour lines depicted in the simplex exhibit similarities with that for pH (Figure 1B), suggesting a common underlying cause for the responses observed in both cases. However, the pattern of the CaCl_2_/KCl relationship is opposed; generally, elevated CaCl_2_ proportions (with low KCl) correlate with increased *firmness*, regardless of the specific MgCl_2_ content associated with a particular ratio. Despite this insight, the challenges encountered during fitting (53% explained variance) imply that *firmness* changes could also depend on other variables not included in the design. Regrettably, the existing literature provides no pertinent references on this specific aspect.

### 3.3. Effect of the Partial Replacement of Salt on the Surface Colour of Olives

The colour of foods is widely recognised as an essential quality attribute. However, the desalting process inevitably leads to a decline in colour quality, primarily affecting the *Ic*, *L*, b*, and *Ch* values, which experienced notable reductions (as indicated in Table 4). Subsequent packaging induced further changes, revealing a semblance of recovery, yet these fluctuations could not be conclusively associated with the concentrations of the chloride salts present in the mixtures. Globally, the colour values closely approximate those observed in the usual packaging practices (Control). This observed behaviour aligns with the experiment’s objectives, which aimed to produce Manzanilla table olives with a colour profile reminiscent of traditional products. Nonetheless, a multivariate analysis might unveil additional relationships not observed in the individual parameter analysis.

### 3.4. Effect of the Partial Replacement of Salt on the Sensory Profile

The results from the evaluation sheet [25] demonstrated that no response surpassed the threshold of 2.5 cm for negative attributes. Therefore, the package olives from all experimental runs were within the First or Fancy category and were suitable and viable commercial products. The average ratings for the remaining descriptors (refer to Table 5) exhibited a relatively narrow range. They closely resembled those of the Control group as the chosen mixtures were intentionally designed to emulate the existing products’ quality closely. Their analysis of the sensory results applying RSM revealed that *appearance* (*p* = 0.55), *smell* (*p* = 0.58), *acid* (*p* = 0.43), and *saltiness* (*p* = 0.59)) were consistently perceived as similar in all packaged olive runs. However, *bitterness*, *firmness*, *fibrousness* and *crunchiness* were significantly related (fit *p*-value ≤ 0.05). In contrast, the lack of fit was insignificant (*p*-value > 0.05) to variations of the salt mixtures in the packaged brines.

For *bitterness*, in addition to significant fit, the model had a precision of 14.7, far above the threshold (4.0) considered adequate for navigating the experimental region. The model was quadratic (linear terms, significant at a *p*-value < 0.0001; interaction CaCl_2_·MgCl_2_ significant at *p* = 0.0195) and took the form:

(4) $$Bitterness=+0.060\cdot \left[KCl\right]+0.426\cdot \left[{CaCl}_{2}\right]+0.380\cdot \left[{MgCl}_{2}\right]-0.023\cdot \left[{CaCl}_{2}\right]\cdot \left[{MgCl}_{2}\right]$$

**Figure 2:**
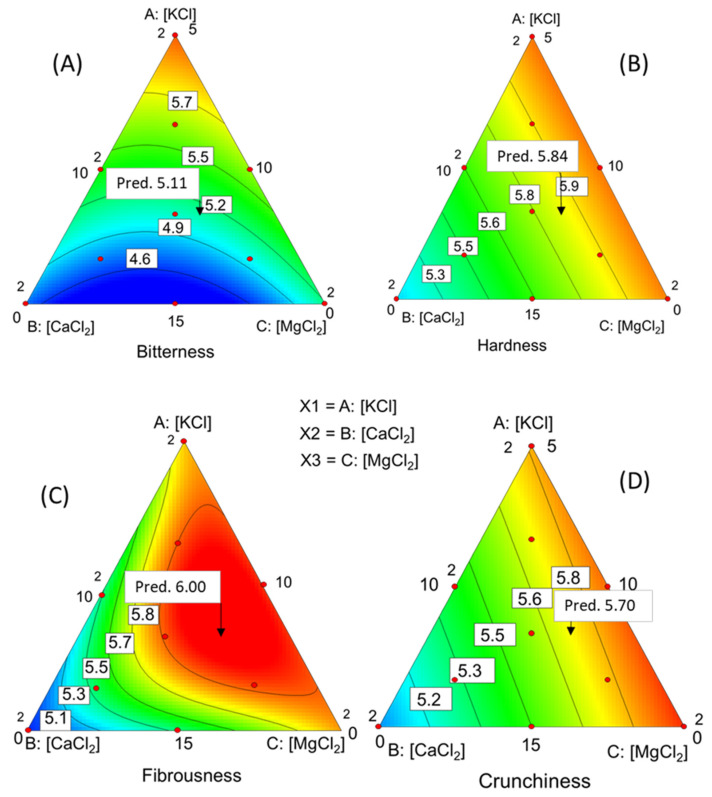
Influence of the CaCl_2_, KCl, and MgCl_2_ (g/L) mixtures in the packaging brines on (**A**) *Bitterness*, (**B**) *Hardness*, (**C**) *Fibrousness*, and (**D**) *Crunchiness*. Pred., predictions at the optimisation point.

CaCl_2_ and MgCl_2_ emerged as the primary contributors to the perception of *bitterness*, albeit with a negative interaction effect between them, potentially due to an interference phenomenon. The plot in the simplex (because of the salts sum constraint) (Figure 2A) unveiled that the contour lines representing bitter scores exhibited an increase as they approached the KCl vertex, where this salt content is the lowest and CaCl_2_ and MgCl_2_ are at the highest concentrations. The contour line inclination towards the right indicates a more pronounced influence of CaCl_2_. Notably, the lowest *bitterness* scores coincide with elevated KCl (aligned with the CaCl_2_-MgCl_2_ axis) levels and approximately half the proportions of the other two salts. The response surface was a descending hill that progressively broadened as the concentration of K increased. Moreover, a steeper descent is observed along the CaCl_2_·KCl border than the MgCl_2_·KCl border. As a result, the graph suggests that CaCl_2_ might contribute to *bitterness* more than MgCl_2_. Additionally, an elevated presence of KCl seems to have a mitigating effect on *bitterness* perception.

The effect of the salt mixtures on the *hardness* sensory scores (Figure 2B) was linear (*p*-value = 0.0008), with a precision of 9.00 (more than double the limit considered enough for the signal-to-noise ratio). Then, the model was adequate to navigate through the experimental region. Its equation in terms of real components was:

(5) $$Hardness\;(score)=+0.206\cdot \left[KCl\right]+0.293\cdot \left[{CaCl}_{2}\right]+0.208\cdot \left[{MgCl}_{2}\right]$$

with CaCl_2,_ the salt that most contributed to *hardness* scoring. When depicted within the simplex (Figure 2B), the visualisation reveals contour lines that exhibit a linear ascent aligned with the CaCl_2_ content, which runs parallel to the border MgCl_2_·KCl, suggesting similar contributions from both MgCl_2_ and KCl to the overall *hardness* score. Notably, this visualisation indicates that the panellists were quite sensible (Figure 2B) in discerning the impact of salt mixtures on *hardness*, surpassing the sensitivity of the instrumental measurements of *firmness* (Figure 1B).

The model for *fibrousness* had high precision (18.00). However, it was somewhat complex since it included linear terms (significant at *p*-value < 0.0001), three-way interaction (significant at *p* = 0.0020), and retained the two-way interactions CaCl_2_·KCl (*p*-value = 0.6308), KCl·MgCl_2_ (*p*-value = 0.2412) and CaCl_2_·MgCl_2_ (*p*-value = 0.765) to make the model hierarchical and adequate for predictions. Then, the equation was:

(6) $$\begin{array}{ll} Fibrousness= & -0.270\cdot \left[KCl\right]-0.701\cdot \left[{CaCl}_{2}\right]-0.870\cdot \left[{MgCl}_{2}\right]+0.1122\\ & \;\cdot \left[KCl\right]\cdot \left[{CaCl}_{2}\right]+0.1183\;\cdot \left[KCl\right]\cdot \left[{MgCl}_{2}\right]+0.169\cdot \left[{CaCl}_{2}\right]\\ & \cdot \left[{MgCl}_{2}\right]-0.01129\cdot \left[KCl\right]\cdot \left[{CaCl}_{2}\right]\cdot \left[{MgCl}_{2}\right] \end{array}$$

which interpretation is only possible after its plotting in the simplex (Figure 2C). The response represents an ascending surface that gains elevation with increasing CaCl_2_ content. It ascends to form a broad plateau around the barycenter and extends up to the KCl·MgCl_2_ boundary. Notably, the surface’s steepest incline is observed at similar concentrations of KCl and MgCl_2_. Interestingly, this “hill” crest follows a trajectory that closely approximates the bisector of the angle formed by the CaCl_2_-KCl and CaCl_2_- MgCl_2_ boundary and exhibits similar slopes at both sides.

Finally, *crunchiness* depended on the levels of salts in the packaging brine through a linear model (*p*-value = 0.0004) whose precision was 10 (far above the limit 4). The equation was:

(7) $$Crunchiness=+0.\;206\cdot \left[KCl\right]+0.288\cdot \left[{CaCl}_{2}\right]+0.191\cdot \left[{MgCl}_{2}\right]$$

The more relevant contributor (highest coefficient) was CaCl_2_. The contour lines were not parallel to the border KCl·MgCl_2_ (Figure 2D) but somewhat inclined, indicating that the surface was a plane slightly climbing from the KCl and CaCl_2_ vertexes towards the MgCl_2_ vertex.

### 3.5. Optimisation

The optimisation had the aim of estimating the proportions of salt mixtures that would lead to an overall desirable outcome (desirability). The criteria for selecting optimal values for the physicochemical, colour, and sensory characteristics were as follows: maximum concentrations of KCl, CaCl_2_, and MgCl_2_ as well as *firmness*, *hardness*, *fibrousness*, and *crunchiness* while minimising pH and *bitterness*. Only one suggestion was obtained, resulting in a desirability value of 0.57. This suggestion consisted of using 12.93 g/L of KCl, 6.94 g/L of CaCl_2_, and 6.38 g/L of MgCl_2_, respectively. Applying these concentrations, the responses regarding the significant variables would be as follows: pH, 3.36, and *firmness*, 19.58 N/g pulp. Additionally, the sensory descriptors would reach the following scores: *bitterness*, 5.11; *hardness*, 5.84; *fibrousness*, 6.00, and *crunchiness*, 5.70. The point of selected concentrations and the predictions for the respective attributes are indicated in each of the previous graphs (Figure 1 and Figure 2).

### 3.6. Multivariate Study of Sensory Attributes, Overall Scoring, and Buying Predisposition

#### 3.6.1. Correlation

A first overview of the scores assigned to the various descriptors hinted at the potential close relationship among some. Employing the Pearson correlation analysis is the most straightforward approach to quantifying such relationships (as depicted in Table 6). Specifically, the *smell* perception was related solely to that of *appearance*. *Acid* scoring was associated with *salty* and *firmness* evaluations. *Bitterness* was linked to all kinesthetic attributes, indicating a robust relationship among these characteristic properties. Furthermore, this alignment suggests a pronounced interdependence between these descriptors. Interestingly, the positive association between *salty* and *acid* or between *hardness*, *fibrousness*, and *crunchiness* could arise due to a lack of clear differentiation among panellists despite their information before testing. It might stem from inherent difficulties in independently appreciating these descriptors.

#### 3.6.2. Sensory Similarities among Treatments

**Figure 3:**
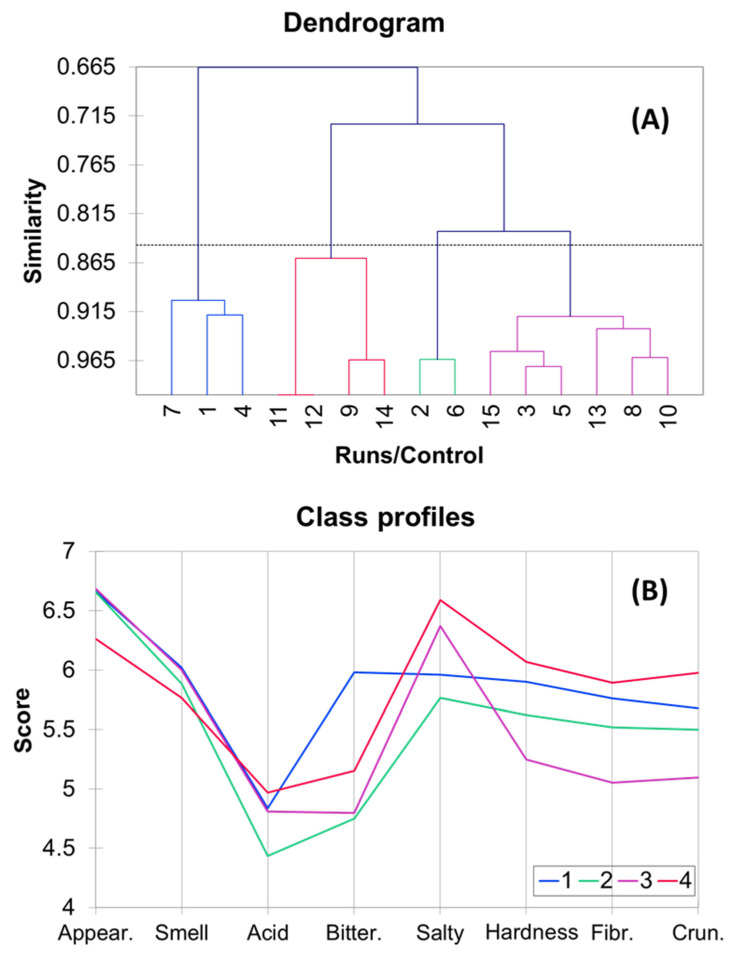
Hierarchical clustering analysis of sensory similarities among variations of CaCl_2_, KCl, and MgCl_2_ combinations. (**A**) Dendrogram illustrating similarities, and (**B**) Class profiles.

Given the fluctuations observed in the scoring of various runs, it becomes valuable to identify salt mixtures leading to similar features. This approach would facilitate the selection of blends resembling traditional products, the most cost-effective alternatives (due to differences in salt costs), or those more suitable for specific markets. The derived dendrogram (Figure 3A,B) introduces several new candidates into class 3 (namely runs 3, 5, 13, 8, and 10, characterised by high proportions of KCl or MgCl_2_). These candidates exhibit proximity to the traditional product (15, Control), displaying high scores for *appearance*, *smell*, and *salty* while lower for kinesthetic sores. Such blends could be promising candidates for initiating salt reduction strategies without significantly modifying consumers’ perceptions.

Intriguingly, other options emerged within the remaining groups, with discernible differences evident in the sensory profiles of each class (Figure 3B). Class 1 (comprising runs 7, 1, and 4) stands out with its high *bitterness* scores (Figure 3A,B), a trait negatively perceived by consumers, as indicated by the correlations (Table 6). Class 4 (runs 11, 12, 9, 14) obtained high *salty* sores. However, the most systematic differences among the classes pertain to the kinesthetic attributes, with the scores in *hardness*, *fibrousness*, and *crunchiness* progressively decreasing from classes 4, 1, 2, and 3 (as observed on the right side of Figure 3B). This research thus provides the industry with valuable insights to choose from a range of formulations.

#### 3.6.3. Study by PLS-R of the Relationship between Sensory Attributes and Overall Score and Buying Predisposition

**Figure 4:**
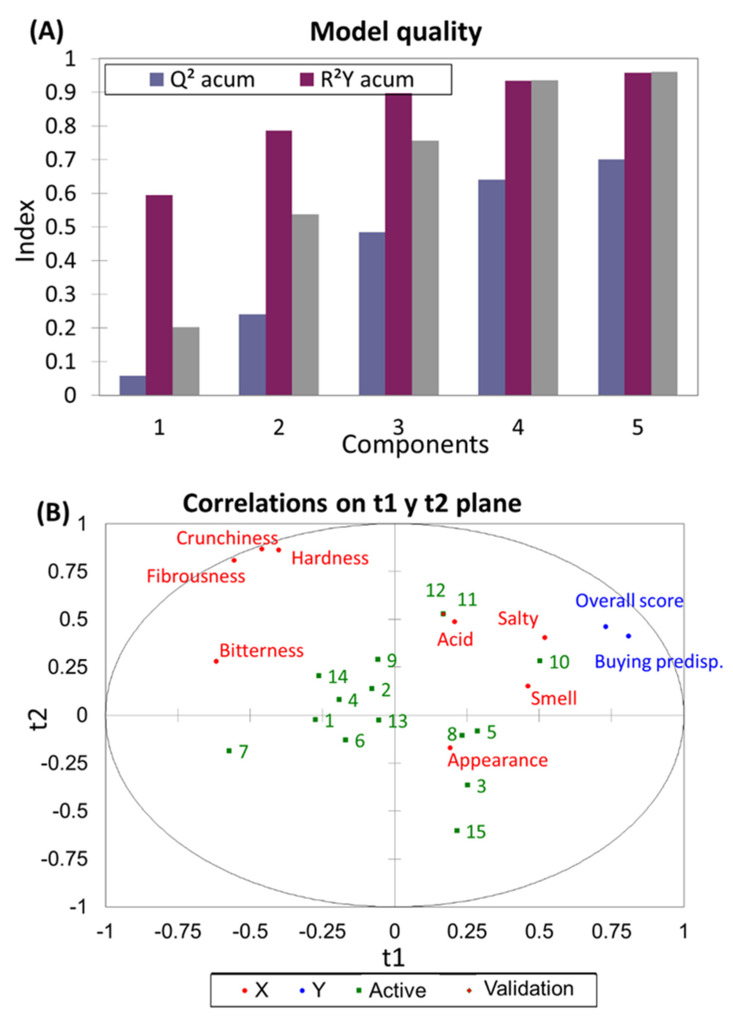
Partial Least Squares-Regression (PLS-R) analysis to explore the connection between sensory attributes, *overall scores*, and *buying predisposition* across the various CaCl_2_, KCl, and MgCl_2_ combinations. (**A**) Model performance assessment; (**B**) Projection of sensory attributes, *overall score*, and *buying predisposition* onto the **t1** and **t2** axes.

Establishing connections between sensory attributes, *overall scores*, and *buying* (or purchasing) *predisposition* can offer valuable insights to the industry for aligning its products with consumers’ preferences. PLS-R analysis was employed to explore such a relationship. A four-components model was deemed satisfactory (Figure 4A), given the adequately explained variance for the dependent variable (R^2^Y Accum., 0.935), independent variable (R^2^X Accum., 0.937), and the overall model quality (Q^2^ Accum., 0.640) appropriate.

Projections onto the t1 and t2 axes (Figure 4B) reveal a positive correlation between the *overall* score and *buying predisposition* with descriptors like *salty*, *acid*, *smell*, and *appearance*. Conversely, negative correlations are observed with descriptors like *bitterness* and kinesthetic traits (excessive *hardness*, *fibrousness*, and *crunchiness*), which are less well-received by consumers. Run 10 was the most appreciated, closely followed by runs 11 and 12. Subsequently, runs 3, 5, 8, and 15 (Control) held favourable positions, possibly due to their association with the traditional product.

**Figure 5:**
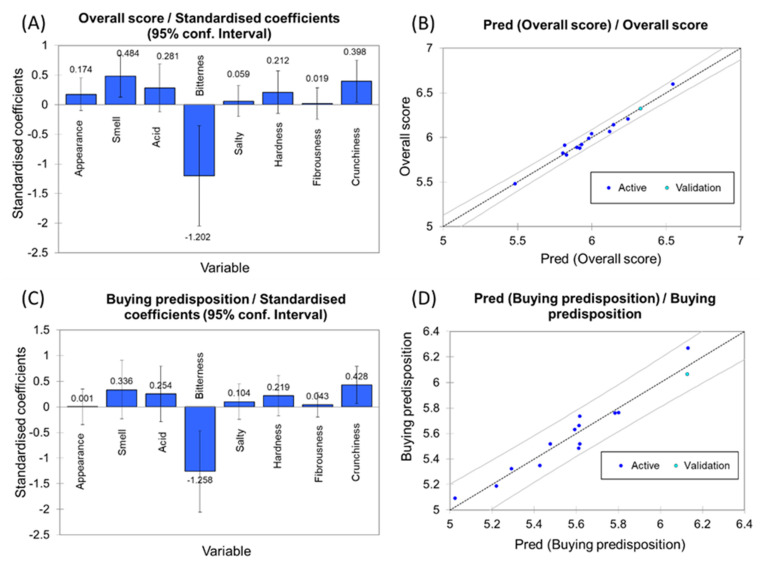
PLS-R investigates the relationship between sensory descriptors, *overall scores*, and *buying predisposition* across the different CaCl_2_, KCl, and MgCl_2_ combinations. (**A**) Standardised model coefficients for *overall scores*; (**B**) Evaluation of *overall score prediction* with 95% confidence interval; (**C**) Standardised model coefficients for *buying predisposition*; (**D**) Evaluation of *buying predisposition prediction*, with 95% confidence interval.

The contribution of each attribute was quantified by its standardised coefficients (Figure 5A,C). Significant contributors to the *overall score* and *buying predisposition* were *bitterness* (highly negative, carrying substantial influence) and *crunchiness* (positively influential). Moreover, for the *overall score*, the descriptor *smell* also held significance. Other descriptors exhibited positive but relatively less impactful contributions. This information then complements the insights from Figure 4B and aids in pinpointing the descriptors that consumers prioritise when evaluating table olives.

The model for obtaining the scores for overall evaluation was:

(8) $$\begin{array}{ll} Overall\;score= & +1.189+0.170\cdot Appearance+0.482\cdot Smell+0.277\cdot Acid-0.521\cdot Bitterness\\ & +\left(4.295\cdot 10^{-2}\right)\cdot Salty+0,110\cdot Hardness+\left(1.127\cdot 10^{-2}\right)\cdot Fibrousness+0.227\\ & \cdot Crunchiness \end{array}$$

which had a determination coefficient of 0.9884 (98.84% explained variance). It is primarily influenced (negatively) by *bitterness*, *smell*, and *crunchiness* (significant coefficients in Figure 5A). It can be used for estimations with a narrow spreading (Figure 5B).

Similarly, the model for estimating *buying predisposition* was:

(9) $$\begin{array}{ll} Buying\;pred.= & +1.997+\left(1.519\cdot 10^{-3}\right)\cdot Appearance+0.406\cdot Smell+0.304\cdot Acid-0.662\cdot Bitterness\\ & +\left(9.139\cdot 10^{-2}\right)\cdot Salty+0,139\cdot Hardness+\left(3.014\cdot 10^{-2}\right)\cdot Fibrousness+0.296\\ & \cdot Crunchiness \end{array}$$

with a determination coefficient of 0.9680 (96.80 explained variance). The PLS-R analysis exhibited negative contributions of *bitterness* and positive for *crunchiness* (Figure 5C). The model can be used to navigate through the experimental region, although with a somewhat higher uncertainty (more comprehensive limits in Figure 5D) than in the *overall score* (Figure 5B).

## 4. Discussion

Most research on reducing NaCl in table olives has primarily concentrated on the fermentation phase. In the context of natural black olive fermentation, Özay and Borcakli [8] reduced the NaCl level from 14 g/100 mL to 6 g/100 mL, primarily focusing on the growth of lactic bacteria and acid production. This reduction increased acidity, reaching up to 0.59 g/100 mL in the test with a reduced level. Tassou [9] investigated even lower levels (4–8%.) and concluded that the best fermentation conditions were achieved at 6% salt concentration and a temperature of 25 °C. This conclusion was based on factors such as free acidity produced and lowest pH, indicating improved fermentation outcomes. Similarly, Kanavouras et al. [10] followed the trend of Özay [8] by substituting sodium with calcium, using readily available Ca(OH)_2_. However, even with this substitution, the best product in colour, texture, and acceptability still had 12.8% salt. Additionally, calcium chloride was found to have a protective effect on the mechanical properties of natural olives [11], which aligns with the traditional empirical use of sodium chloride in Spanish style [2]. Subsequently, Panagou et al. [13] explored the impact of different salt mixtures of NaCl, KCl and CaCl_2_ on the fermentation profiles of natural black *Conservolea* olives. They concluded that only combining KCl and NaCl resulted in olives with favourable organoleptic properties. This suggests that a specific balance of these salts is crucial for achieving desirable sensory characteristics and texture during these products’ fermentation process.

In other styles, for instance, Zinno et al. [34] replaced 25%, 50%, and 75% NaCl with KCl during the fermentation of Nocellara del Belice olives processed as Spanish and Castelvetrano styles while maintaining a final saline concentration of 9 and 7%, respectively. Interestingly, their findings indicated that these substitutions did not significantly impact microbial dynamic, contamination risk, or proliferation of pathogens or spoilage microorganisms. Another study by Saúde et al. [35] involved the fermentation of Maçanilha Algarvia olives with partially substituting NaCl using KCl and CaCl_2_, resulting in an overall 8% salt concentration. The evaluation by the sensory panel noted that olives fermented in 8% NaCl and 4%NaCl + 4%KCl exhibited the most favourable flavour and general attributes. This formulation also yielded a remarkable 672% increase in K content and a simultaneous 19% reduction in Na. In another research, Bautista Gallego et al. [12] applied salt substitution in cracked *Aloreña de Málaga* olives that were directly brined. Their findings showed that using CaCl_2_ reduced the growth of Enterobacteriaceae and lactic acid bacteria but resulted in heightened yeast activity. Furthermore, the process was accompanied by decreased pH and combined acidity, as in the current study.

Similarly, the application of a mixture of the same salts during the fermentation of green Spanish-style Gordal olives [14] demonstrated that CaCl_2_ impacted initial and post-fermentation pH values, delayed sugar diffusion into the brine, and produced a higher titratable acidity concentration. Concurrently, incorporating KCl during this fermentation process reduced the growth of Enterobacteriaceae and yeast, promoted the proliferation of lactic acid bacteria, and led to the lowest pH, which could be helpful for the further preservation of the packaged olives. Regarding sensory attributes, the *saltiness* perception was associated with NaCl and KCl concentrations, while *bitterness*, *hardness*, *fibrousness* and *crunchiness* were linked to the presence of CaCl_2_ [15]. Most of these observations were consistent with the changes described in our packaged olives, except for microbial growth.

During packaging, this study successfully replaced NaCl with KCl, CaCl_2_, and MgCl_2_ mixtures in plain (whole) green table olives. This strategy eliminated the potential risks associated with low salt levels during processing and was exclusively applied to the ready-to-consume, typically pasteurised product. While the initial desalting process caused a noticeable rise in pulp moisture, this effect was subsequently reduced to levels similar to Control. Furthermore, substituting salt-induced modifications in the physicochemical characteristics resulted in a decrease in pH, a slight uptick in titratable acidity, and an enhancement of olive *firmness*, primarily attributed to the inclusion of CaCl_2_. The reduced combined acidity observed in the final products was mainly caused by the desalting process, compounded by the dilution effect inherent in the packaging phase. The investigation also reaffirmed that the combined acidity primarily originates from lactic salts. This deduction was supported by the close agreement between the lactic estimation, derived from both titratable acidity and combined acidity as lactate. Additionally, the data revealed that lactic acid concentrations in the pulp moisture closely paralleled those in the brine, indicating that lactic acid is nearly exclusively accumulated in the pulp moisture.

Interestingly, the introduction of salt mixtures did not cause a discernible impact on colour, as the experimental design aimed to replicate the traditional commercial presentations of the product closely. Regarding sensory descriptors, the presence of CaCl_2_ significantly elevated sores for *bitterness*, *hardness*, *fibrousness*, and *crunchiness*. Conversely, MgCl_2_ exerted only a slight influence on the sensory descriptors. Notably, the effect of CaCl_2_ on *firmness* or *hardness* (as perceived by sensory evaluation) aligns with findings from previous research [36,37]. In contrast, the incorporation of KCl reduced the *bitterness* perception. However, beyond the inherent olive *bitterness,* the exact mechanism by which other elements contribute to *bitterness* remains elusive.

In theory, RSM should lead to conditions that produce the best response. However, the situation becomes more complex when several variables with multiple impacts are involved, as in this case where desirability only reached 0.57. Regarding the concentration of salt mixtures for achieving overall optimum results, the selection prioritised KCl at a higher level. This choice is crucial as it can compensate for the substantial loss of potassium during the green Spanish-style olive processing [15]. Calcium chloride also received a high priority and surely will increase the natural content of the olive fruit in Ca [2,3]. Finally, Mg is not abundant in table olives but its incorporation could be interesting for improving their nutritional profile [4]. Regarding the effect of salts on the physicochemical characteristics and colour, it is worth noting that the predicted pH at the optimum point may be not the most desirable in typical packaging since other combinations could yield the lowest values. However, such a condition is not as critical under pasteurisation stabilisation. Notably, the *firmness* is relatively close to maximum values and the selection could be quite appropriate for this attribute. In the case of sensory descriptors, *bitterness* resulted in somewhat unfavourable treatment, due to the low proportion of potassium, which appears to mitigate this sensation. This happened despite having given *bitterness* the maximum weight in the selection criteria. Notably, the selection is highly favourable for *fibrousness* since the chosen point aligns with the maximum scoring region. Furthermore, *hardness* and *crunchiness* approach their maximum values. In summary, the selection was generally appropriate, except for *bitterness*, which was challenging to minimise. To improve consumer acceptance, it might be beneficial to adjust the selected composition towards the centre of the design zone with lower *bitterness*. This supports the notion that, in the case of multiple variables with various impacts, further refinement may be necessary.

Bansal and Rani [38] have attributed the limited adoption of potassium chloride as a substitute for NaCl in lemon pickles to its tendency to impart a bitter taste. This bitter perception of KCl has been observed in meat products like fermented sausages and other applications. In the context of NaCl substitution with KCl, it has been noted that a maximum of 16% substitution is feasible to prevent the development of *bitterness* perception, apparently caused by the K cation [39]. Youssef et al. [40] studied the production of low-sodium pickles suitable for hypertensive patients, exploring binary and ternary mixtures. The research found that a blend of 4%NaCl + 4%KCl resulted in negligible changes in taste and overall acceptability compared to 8%NaCl formulations. While the *firmness* of cucumbers was reduced, carrots exhibited a minor increase. However, no *bitterness* increase was reported.

Regarding table olives, Ambra et al. [41] investigated the partial replacement of NaCl with KCl (at levels of 50% or 75%) in brine used during the fermentation of Spanish and Castelvetrano styles. This substitution led to a product with significantly reduced sodium content without substantially affecting the presence of the bioactive compounds. However, introducing KCl was associated with a heightened *bitterness* in both debittering methods. Despite this effect, the *bitterness* perception, persistence and aftertaste in the Castelvetrano system olives remained below that of the classic Spanish style, as the latter retained better the characteristic “sweet olives”. Erdogan et al. [42] studied five combinations involving NaCl, CaCl_2_, and KCl to partially replace sodium in table olive production via the traditional Gemlik method. The sensory analysis revealed that mixtures containing only CaCl_2_ and KCl produced bitter olive products. In contrast, combinations containing 5% NaCl and 5% KCl yielded low sodium olives with satisfactory organoleptic characteristics, aligning with the outcomes of our current research. In recent experiments conducted with the Turkish *Sari Ulak* cv. cracked table olives [16], the preference was given to olives processed with a combination of NaCl and KCl over those processed using CaCl_2_ alone or in combination with NaCl. Consequently, the precise impact of CaCl_2_ and KCl salts on sensory characteristics after NaCl replacement remains unclear. Nevertheless, including KCl in brines can yield acceptable low-sodium table olives.

Our study further highlighted a robust relationship among kinesthetic attributes. Nonetheless, despite detailed explanations, discerning between *firmness*, *fibrousness*, and *crunchiness* remained intricate for testers. This challenge, however, is general and has been observed in previous research [15]. Despite its complexity, *crunchiness* is valuable and distinguishes certain presentations, such as *Aloreña de Málaga* [12].

The positive correlation between *acid*, *salty*, and *firmness* suggests a parallel trend. However, it might also indicate confusion in distinguishing between *acid* and *salty*. The outcomes in Figure 1B and Figure 2B support earlier findings concerning *Gordal* olives [14]. Interestingly, our results suggest that the most pronounced bitter scores materialise without KCl, implying that KCl might not significantly contribute to *bitterness*. Conversely, an increase in MgCl_2_ seems to intensify the perception of *bitterness*.

In contrast, CaCl_2_ was associated with *firmness* and *crunchiness* scores, with their peak values observed at minimal proportions of KCl and MgCl_2_. This correlation between CaCl_2_ and *bitterness* and kinaesthetic attributes also finds support in the research conducted by several authors, utilising an enlarged centroid mixture design. These researchers discovered that elevating CaCl_2_ concentrations enhanced a range of properties, encompassing attributes like *Ic*, *L* and *b* * values, *firmness*, *bitterness*, *hardness*, *fibrousness*, *crunchiness*, and *salty* (negatively), as assessed through PLS-R analysis. Most treatments exhibited healthier properties (highly favourable mineral nutrients), resembling commercial products. Our study similarly determined that *bitterness* was associated with all kinaesthetic attributes. This correlation is logical, given that elevated scores in these attributes may indicate less mature olive or mild lye treatment, leading to more pronounced *bitterness* in the fruits. Nonetheless, a comprehensive understanding of the changes in *bitterness* is still in progress, possibly due to the intricate interplay of the various salts involved in the experiments. Then, this aspect remains a subject still requiring further research.

The identification that *bitterness* had a noteworthy impact on the overall scoring and *buying predisposition* is outstanding. Conversely, higher ratings in *smell* and *crunchiness* corresponded to improved *overall scoring* and a greater inclination to purchase. The categorisation of runs into distinct sensory profiles through clustering based on sensory attributes can, in turn, guide their choices by processors based on consumer preferences or specific market demands.

## 5. Conclusions

Employing RSM, the primary objective of this investigation was to examine the effects of partial (50%) replacement of NaCl with KCl, CaCl_2_ and MgCl_2_ (considered controlled variables) in the packaging brine on the characteristics (the responses) of plain green Spanish-style Manzanilla olives. Applying pasteurisation to stabilise the products reduced any possible safety risk derived from the substitution. CaCl_2_ exhibited the most significant effect on the final products among the various salts tested. As CaCl_2_ levels increased in the brine, there was a linear decrease in pH and an increase in *firmness, hardness, and crunchiness.* The *bitterness* model incorporated an interaction between CaCl_2_ and MgCl_2_, resulting in a minimum point around their respective half concentrations and maximum KCl. Regarding *fibrousness*, the model revealed significant linear and cubic terms, leading to an area of top scores from the barycenter of the design to the maximum CaCl_2_ levels, which extended around the central point (half concentrations) of the KCl-MgCl_2_ axis. This indicates the presence of a saturating effect beyond two-thirds of its content range. In contrast, an increase in KCl was related to reduced *bitterness*. Contrary to other studies, our results demonstrated that introducing KCl in the presence of CaCl_2_ and MgCl_2_ can mitigate the bitter sensation. Furthermore, optimisation resulted in the choice of a combination with relatively low desirability (0.57). This combination should be refined, especially for markets that demand lower *bitterness* levels, which could be achieved by increasing the KCl content or reducing the CaCl_2_ level in the packaging brines.

PLS-R analysis indicated that the pivotal attributes influencing *overall appreciation* were *smell* and *crunchiness* while *buying predisposition* was promoted by *crunchiness*. Conversely, *bitterness* had a detrimental impact on these appreciations. The study also facilitated the classification of salt mixtures with similar profiles, which could assist processors in selecting products based on consumers’ demands or specific collective targets.

## Figures and Tables

**Table 1:** Experimental design of basic lattice mixture for partial (50%) replacement of NaCl in the final packaging of green Spanish-style Manzanilla table olives. Levels in the customary units used in table olives technology.

Design Point (Runs)	Chloride Salts in the Mixture (g/L)		
	KCl	CaCl_2_	MgCl_2_
1	5	10	10
2	15	10	0
3	15	0	10
4	5	10	10
5	10	5	10
6	15	5	5
7	8.33	8.33	8.33
8	15	0	10
9	15	10	0
10	13.33	3.33	8.33
11	13.33	8.33	3.33
12	11.67	6.67	6.67
13	10	5	10
14	10	10	5

**Table 2:** Colour index scale and its correspondence with the sensory evaluation by a panel for green Spanish-style Manzanilla table olives.

Ic Interval	Panel Evaluation
33.6–30.2	Excellent
30.2–26.8	Good
26.8–23.7	Acceptable
23.7–21.0	Bad
<21.0	Very bad

**Table 3:** Physicochemical characteristics of the stored green Spanish-style Manzanilla brines and fruits (raw material). The table also includes the desalting solution, the packaged olives (runs) at the end of the equilibrium period, and the usual packaging as Control.

Treatment	Brines					Olives		
	pH	Titratable Acidity (g/L)	Combined Acidity (mEq/L)	Estimated Lact. ^3^ (g/L)	Lact. (g/L)	Lact. in Pulp Moisture (g/L)	Moisture in Pulp (g/100 g)	Firmness (N/g)
Storage product	3.89 (0.03)	7.07 (0.17)	82.5 (1.8)	14.49 (0.31)	9.86 (0.07)	10.63 (0.01)	69.23 (0.28)	17.98 (0.17)
Desalting solution ^1^	3.93 (0.05)	2.90 (<0.01)	40.4 (0.5)	6.54 (0.05)	1.35 (0.01)	3.30 (0.01)	76.00 (0.04)	13.23 (2.60)
Run 1	3.20 (0.09)	2.80 (0.40)	14.2 (1.7)	4.07 (0.25)	3.69 (0.22)	4.13 (0.01)	74.38 (0.38)	22.19 (0.14)
Run 2	3.46 (0.03)	2.60 (<0.01)	21.5 (2.8)	4.53 (0.25)	4.30 (0.02)	4.38 (0.02)	74.06 (0.12)	19.14 (1.26)
Run 3	3.89 (0.01)	1.50 (<0.01)	22.1 (0.2)	3.49 (0.02)	2.66 (0.11)	2.55 (0.13)	73.26 (0.37)	15.89 (0.27)
Run 4	3.37 (0.03)	3.00 (0.20)	20.6 (0.8)	4.86 (0.27)	4.18 (0.04)	4.37 (0.08)	72.62 (0.70)	19.98 (0.14)
Run 5	3.30 (0.03)	3.15 (0.25)	16.7 (0.5)	4.65 (0.20)	4.54 (0.22)	4.82 (0.05)	73.94 (0.03)	19.79 (0.17)
Run 6	3.35 (0.01)	2.80 (<0.01)	17.4 (1.0)	4.37 (0.09)	4.94 (0.08)	4.84 (0.02)	73.49 (0.44)	17.26 (0.14)
Run 7	3.26 (0.01)	3.10 (0.10)	15.2 (0.1)	4.69 (0.09)	3.67 (0.11)	3.95 (0.06)	76.11 (0.12)	16.32 (0.04)
Run 8	3.44 (0.02)	2.50 (<0.01)	24.4 (0.3)	4.69 (0.03)	5.21 (0.03)	5.30 (0.10)	73.91 (0.10)	15.39 (0.16)
Run 9	3.40 (0.01)	3.30 (<0.01)	20.9 (1.0)	5.18 (0.09)	5.40 (0.08)	5.50 (0.13)	75.05 (0.51)	18.64 (0.09)
Run 10	3.49 (0.03)	2.95 (0.05)	24.2 (1.7)	5.12 (0.10)	5.43 (0.11)	5.45 (0.07)	74.71 (0.22)	17.55 (0.10)
Run 11	3.34 (0.03)	2.65 (0.05)	22.0 (0.1)	4.63 (0.06)	5.67 (0.07)	5.45 (<0.01)	73.56 (0.23)	22.72 (0.04)
Run 12	3.27 (0.02)	3.25 (0.05)	20.5 (0.3)	5.10 (0.02)	5.63 (0.10)	5.68 (0.07)	74.82 (0.04)	21.41 (0.09)
Run 13	3.31 (0.03)	2.75 (0.05)	22.5 (0.4)	4.78 (0.02)	5.60 (0.13)	5.49 (0.15)	73.40 (0.40)	22.00 (0.04)
Run 14	3.31 (0.02)	3.30 (<0.01)	21.2 (0.1)	5.20 (0.01)	5.64 (0.01)	5.56 (0.07)	73.97 (0.30)	20.71 (0.03)
Control ^2^	3.61 (0.05)	2.80 (<0.01)	24.9 (0.2)	5.04 (0.01)	4.73 (0.12)	4.67 (0.16)	75.60 (0.63)	15.96 (0.10)

Notes: Standard error in the parenthesis. Lact. Lactic acid; ^1^ Desalted up to 2.5% NaCl in pulp moisture; ^2^ Packaged with only NaCl and 0.5% lactic acid in equilibrium; ^3^ Considering that titratable and combined acidities were from lactic acid. For detailed explanations regarding the combinations of chloride salts used in each run, refer to Table 1.

**Table 4:** Colour profile of the stored green Spanish-style Manzanilla fruits (raw material). The table also includes the desalted olives, the packaged olives (runs) at the end of the equilibrium period, and the usual packaging as Control.

Treatment	*I_C_*	L*	A*	B*	*Ch*	*h*	(–a*/b*)
Storage olives	27.45 (0.36)	51.20 (0.18)	4.85 (0.13)	35.90 (0.67)	36.23 (0.68)	82.30 (0.13)	−0.135 (0.005)
Desalted olives ^1^	25.67 (1.61)	46.64 (1.44)	4.58 (0.06)	31.91 (0.83)	32.23 (0.81)	81.83 (0.31)	−0.143 (0.005)
Run 1	25.98 (0.30)	50.09 (0.07)	4.76 (0.26)	34.37 (0.83)	34.70 (0.86)	82.12 (0.24)	−0.138 (0.004)
Run 2	25.03 (0.90)	48.91 (0.57)	4.63 (0.09)	31.79 (0.39)	32.12 (0.39)	81.72 (0.05)	−0.146 (0.001)
Run 3	25.36 (0.14)	49.47 (0.27)	4.60 (0.02)	33.44 (0.87)	33.75 (0.86)	82.17 (0.23)	−0.138 (0.004)
Run 4	26.75 (0.17)	50.78 (0.78)	4.66 (0.17)	34.12 (0.01)	34.43 (0.31)	82.22 (0.28)	−0.137 (0.005)
Run 5	25.92 (0.22)	49.72 (0.42)	4.88 (0.04)	33.61 (0.28)	33.97 (0.29)	81.74 (0.00)	−0.145 (0.000)
Run 6	24.69 (0.34)	47.63 (0.03)	5.50 (0.17)	32.08 (0.28)	32.55 (0.30)	80.28 (0.20)	−0.171 (0.004)
Run 7	25.23 (0.38)	48.40 (0.46)	5.13 (0.02)	31.44 (0.17)	31.76 (0.12)	80.70 (0.01)	−0.164 (0.001)
Run 8	27.07 (0.32)	50.33 (0.25)	4.75 (0.07)	34.80 (0.23)	35.12 (0.24)	82.23 (0.07)	−0.150 (0.003)
Run 9	25.40 (0.51)	48.97 (0.16)	4.92 (0.06)	32.72 (0.33)	33.09 (0.34)	81.45 (0.02)	−0.148 (0.005)
Run 10	24.84 (0.09)	48.54 (0.33)	4.95 (0.16)	33.43 (0.15)	33.80 (0.12)	81.58 (0.31)	−0.134 (0.003)
Run 11	25.31 (0.62)	49.60 (0.10)	4.58 (0.06)	34.15 (0.38)	34.46 (0.37)	82.36 (0.18)	−0.137 (0.004)
Run 12	25.75 (0.57)	49.94 (0.88)	4.84 (0.16)	35.25 (0.14)	35.58 (0.16)	82.18 (0.22)	−0.150 (0.004)
Run 13	25.27 (0.32)	49.06 (0.37)	4.94 (0.25)	33.00 (0.96)	33.64 (0.91)	81.48 (0.66)	−0.150 (0.012)
Run 14	24.98 (0.25)	48.79 (0.29)	5.05 (0.06)	34.23 (0.56)	34.60 (0.54)	81.61 (0.22)	−0.147 (0.004)
Control ^2^	25.06 (0.06)	48.31 (0.20)	5.05 (0.06)	32.21 (0.44)	32.60 (0.43)	81.10 (0.22)	−0.157 (0.004)

Notes: Standard error in parenthesis: ^1^ Desalted up to 2.5% NaCl in pulp moisture; ^2^ Packaged with only NaCl and 0.5% lactic acid in equilibrium. For explanations concerning run combinations, see Table 1.

**Table 5:** Mean score (standard error in bracket) from Descriptive Quantitative Analysis conducted on the olives from the various runs with 50% NaCl substituted by KCl, CaCl_2_, and MgCl_2_. Comparative data of the usual packaging (Control) is also provided for reference.

Treatment	*Appearance*	*Smell*	*Acid*	*Bitterness*	*Salty*	*Hardness*	*Fibrousness*	*Crunchiness*	*Overall Scoring*	*Buying Predisposition*
Run 1	6.73 (0.19)	6.29 (0.20)	4.97 (0.22)	6.12 (0.24)	5.81 (0.20)	5.89 (0.18)	5.73 (0.18)	5.68 (0.21)	5.82 (0.17)	5.19 (0.19)
Run 2	7.00 (0.17)	6.07 (0.19)	4.57 (0.19)	4.98 (0.20)	5.78 (0.19)	5.85 (0.15)	5.72 (0.17)	5.77 (0.17)	6.20 (0.17)	5.76 (0.19)
Run 3	6.71 (0.18)	5.99 (0.20)	4.80 (0.20)	5.03 (0.22)	6.62 (0.18)	4.91 (0.17)	4.90 (0.18)	4.82 (0.18)	5.89 (0.18)	5.35 (0.20)
Run 4	6.78 (0.18)	6.08 (0.18)	5.20 (0.20)	6.16 (0.23)	6.28 (0.17)	6.17 (0.17)	5.77 (0.17)	5.79 (0.19)	5.80 (0.18)	5.32 (0.18)
Run 5	7.07 (0.17)	6.22 (0.18)	4.93 (0.20)	5.28 (0.23)	6.58 (0.18)	5.56 (0.19)	5.20 (0.17)	5.25 (0.19)	6.14 (0.18)	5.52 (0.19)
Run 6	6.32 (0.19)	5.70 (0.19)	4.30 (0.21)	4.52 (0.20)	5.76 (0.17)	5.39 (0.17)	5.31 (0.17)	5.22 (0.19)	5.92 (0.18)	5.48 (0.20)
Run 7	6.47 (0.17)	5.69 (0.19)	4.34 (0.22)	5.66 (0.25)	5.79 (0.21)	5.64 (0.17)	5.79 (0.44)	5.57 (0.18)	5.48 (0.19)	5.09 (0.21)
Run 8	6.40 (0.19)	5.81 (0.18)	4.81 (0.23)	4.51 (0.22)	6.38 (0.16)	5.35 (0.44)	4.97 (0.16)	5.03 (0.18)	6.06 (0.17)	5.76 (0.19)
Run 9	6.31 (0.19)	5.72 (0.19)	4.92 (0.22)	5.45 (0.25)	6.78 (0.16)	6.06 (0.18)	5.83 (0.17)	5.95 (0.19)	5.88 (0.17)	5.66 (0.18)
Run 10	6.81 (0.19)	6.48 (0.20)	4.80 (0.22)	4.72 (0.21)	6.47 (0.16)	5.71 (0.18)	5.43 (0.16)	5.47 (0.18)	6.59 (0.15)	6.27 (0.17)
Run 11	6.14 (0.18)	5.88 (0.18)	5.03 (0.22)	4.80 (0.23)	6.55 (0.16)	6.03 (0.16)	5.93 (0.18)	5.98 (0.17)	6.32 (0.16)	6.06 (0.18)
Run 12	6.19 (0.17)	5.68 (0.17)	5.07 (0.19)	5.24 (0.19)	6.50 (0.16)	5.69 (0.14)	5.48 (0.16)	5.55 (0.16)	6.04 (0.15)	5.71 (0.16)
Run 13	6.62 (0.19)	5.71 (0.20)	5.02 (0.22)	5.16 (0.20)	6.27 (0.17)	5.59 (0.17)	5.43 (0.17)	5.57 (0.17)	5.99 (0.16)	5.63 (0.17)
Run 14	6.46 (0.17)	5.58 (0.18)	4.89 (0.20)	5.56 (0.22)	6.50 (0.20)	6.16 (0.16)	5.88 (0.17)	6.00 (0.16)	5.91 (0.16)	5.52 (0.18)
Control ^1^	6.50 (0.18)	5.79 (0.18)	4.49 (0.21)	4.07 (0.18)	5.91 (0.17)	4.36 (0.15)	4.38 (0.05)	4.45 (0.17)	6.04 (0.19)	5.73 (0.19)

Notes: Standard error in parenthesis, ^1^ Packaged using solely NaCl and 0.5% lactic acid at equilibrium. For detailed explanations regarding the combinations of chloride salts used in each run, refer to Table 1.

**Table 6:** Correlation among the descriptors’ scores given by the panellists, including the overall scoring and buying predisposition, of the green Spanish style table olives packaged in brines with NaCl partially substituted (50%) with KCl, CaCl_2_, and MgCl_2_.

	*Appearance*	*Smell*	*Acid*	*Bitterness*	*Salty*	*Hardness*	*Fibrousness*	*Crunchiness*	*Overall Scoring*	*Buying Predisposition*
*Appearance*	1	0.652 *	0.04	0.3	−0.168	−0.095	−0.211	−0.211	0.034	−0.252
*Smell*	0.652 *	1	0.281	0.167	0.038	0.083	−0.023	−0.037	0.469	0.193
*Acid*	0.04	0.281	1	0.432	0.656 *	0.530 *	0.365	0.468	0.247	0.191
*Bitterness*	0.3	0.167	0.432	1	0.002	0.625 *	0.601 *	0.550 *	−0.547 *	−0.633 *
*Salty*	−0.168	0.038	0.656 *	0.002	1	0.248	0.118	0.218	0.362	0.405
*Hardness*	−0.095	0.083	0.530 *	0.625 *	0.248	1	0.950 *	0.966 *	0.058	0.072
*Fibrousness*	−0.211	−0.023	0.365	0.601 *	0.118	0.950 *	1	0.979 *	−0.038	0.012
*Crunchiness*	−0.211	−0.037	0.468	0.550 *	0.218	0.966 *	0.979 *	1	0.064	0.124
*Overall scoring*	0.034	0.469	0.247	−0.547 *	0.362	0.058	−0.038	0.064	1	0.929 *
*Buying predisposition*	−0.252	0.193	0.191	−0.633 *	0.405	0.072	0.012	0.124	0.929 *	1

Note: Significant correlations at ≤0.05 are marked with an asterisk. Freedom degrees, 15.

## Data Availability

Data is contained within the article.
